# Immunotherapeutic approaches to ovarian cancer treatment

**DOI:** 10.1186/s40425-015-0051-7

**Published:** 2015-03-24

**Authors:** Cariad Chester, Oliver Dorigo, Jonathan S Berek, Holbrook Kohrt

**Affiliations:** Department of Medicine, Division of Oncology, Stanford University School of Medicine, Stanford, CA USA; Stanford Women’s Cancer Center, Division of Gynecologic Oncology, Department of Obstetrics and Gynecology, Stanford University School of Medicine, Stanford, CA 94305 U.S.A

**Keywords:** Ovarian cancer, ID8, Antibody, TAM, Immune checkpoint blockade, IDO, Cancer vaccine, ACT

## Abstract

Despite advances in combinatorial chemotherapy regimens and the advent of intraperitoneal chemotherapy administration, current therapeutic options for ovarian cancer patients are inadequate. Immunotherapy offers a novel and promising therapeutic strategy for treating ovarian tumors. Following the demonstration of the immunogenicity of ovarian tumors, multiple immunotherapeutic modalities have been developed. Antibody-based therapies, immune checkpoint blockade, cancer vaccines, and chimeric antigen receptor-modified T cells have demonstrated preclinical success and entered clinical testing. In this review, we discuss these promising immunotherapeutic approaches and emphasize the importance of combinatorial treatment strategies and biomarker discovery.

## Introduction

In 2012, there were an estimated 239,000 new cases of ovarian cancer worldwide leading to over 140,000 deaths [1]. Epithelial ovarian cancer is the fourth most common cause of cancer-related death in women in the developed world, where it is also the leading cause of death from gynecological malignancies [2]. The lethality of ovarian cancer is in part due to the difficulty of early detection. Ovarian cancer causes few perceptible symptoms when localized to the ovary. Due to the asymptomatic nature of early disease, most patients do not seek medical care until the disease has progressed beyond the ovaries into the abdomen and pelvis [3]. Nearly 75% of patients present with stage III and IV ovarian cancer [4]. Management of ovarian cancer primarily includes cytoreductive surgery and platinum-based chemotherapy. While clinical remissions are obtainable, the majority of patients will relapse and die of disease, with a 5-year survival of approximately 30% [5]. Novel therapies need to be integrated into ovarian cancer treatment strategies to achieve durable clinical outcomes.

In the last two decades, advances in the understanding of ovarian cancer immunogenicity have opened the door to immunotherapeutic approaches to ovarian cancer treatment. A crucial early step in establishing the validity of ovarian cancer immunotherapy was the observation that CD3^+^ tumor-infiltrating T cells correlated with increased overall survival [6]. Later work confirmed the importance of tumor-infiltrating lymphocytes (TILs) and specifically identified the CD3^+^, CD8^+^ T cells as important antitumor effectors [7]. The identification of tumor-associated antigens (TAAs) lent additional support to an immunotherapeutic treatment strategy. TAAs aberrantly up-regulate in tumor tissue and ascites of ovarian cancer patients and include members of the cancer-testis antigen family (e.g. MAGE-A4 and NY-ESO-1), growth-activating receptors (e.g. HER2/neu), folate receptor alpha (FRα), p53, and CA125 [8-10]. These markers are potential therapeutic targets for eliciting an immune response specific to ovarian cancer and effecting immune-mediated tumor rejection. The Food and Drug Administration (FDA) has approved immunotherapies for prostate cancer, advanced kidney cancer, lymphoma, and metastatic melanoma, but only recently have immunotherapies targeting ovarian cancer entered clinical testing (Table 1). In this review, we discuss advances in immunotherapeutic approaches to ovarian cancer. We divide therapeutic strategies into four categories: antibodies, immune checkpoint inhibitors, vaccines, and adoptive cell therapy (ACT).

**Table 1:** **Key trials in ovarian cancer immunotherapy**

**Study**	**Patients**	**Treatment**	**# of Patients**	**Response**	**Survival**
Vald, Edwards *Cancer Immunol Immunother 2010*	Recurrent, platinum resistant ovarian cancer	Interleukin-2 i.p.	24	CR: 4	Median Survival:
		6 × 10^5^ IU/ml weekly × 16		PR: 2	Non-Responder:
				SD: 7	1.5 years
					Responders: not reached
					(24 -120+ months)
Hodi, Dranoff	Recurrent metastatic ovarian cancer	CTLA-4 Blockade:	9	CR: 0	Duration of Response:
*PNAS 2008*		Ipilupimab i.v. 3 mg/kg q 2 – 3 months		PR: 1	SD: 2,4,6+ Months
				SD: 3	PR: 35+ Months
Diefenbach, Dupont	“High Risk” ovarian cancer after surgery and 1^st^ line chemo	NY-ESO-1b peptide (position 157–165; 100 μg) + 0.5 mL Montanide ISA-51 s.c. q 3 weeks × 5	9	NA	Median PFS: 13 months
*Clin Cancer Res 2008*					6/9 patients recurred
					3 patient disease free after 25, 38, and 52
Fujita,Tanaka *Clin Cancer Research 1995*	NED after surgery and 1^st^ line chemo	1.0 – 4.4 × 10^9^ TIL after 1^st^ line chemo	13 TIL	NA	3-year DFS:
			11 Control		TIL: 82.1%
					Control: 54.5%
					3-year DFS, residual disease after surgery
					TIL: 76.2%
					Control: 33.3%
Chu, June	Consolidation after 1^st^ line treatment or secondary debulking	Her-2/neu, hTERT, PADRE-loaded Dendritic cells +/− Cyclophosphamide	11	NA	5 recurrences
*Cancer Immunol Immunother 2012*					6 patients NED at 36 months
					3-year OS 90%
Odunsi, Jaeger	Consolidation after 1^st^ line treatment	I.d. rV-NY-ESO-1,	22	NA	PFS: 21 months
*PNAS 2012*		3.1 × 10^7^ PFU, monthly s.c.			OS: 48 months
		rF-NY-ESO-1, 7.41 × 10^7^ PFU for 6 mo.			
Rahma, Khleif	HLA-A2.1 + stage III, IV, or recurrent ovarian	Arm A: s.c. wt p53:264–272 peptide with Montanide and GM-CSF.	21	NA	Arm A:
*Cancer Immunol Immunother 2012*					Immune responses 9/13 patients (69%), PFS: 4.2 months
	Cancer over-expressing the p53 protein, no evidence of disease	Arm B: i.v. wt p53:264–272 peptide-pulsed dendritic cells IV			OS: 40.8 months
		IL-2 in both cohorts			Arm B:
					Immune responses 5/6 patients (83%)
					PFS: 8.7 months
					OS: 29.6 months

Adapted from: Kandalaft LE, Powell DJ Jr, Singh N, Coukos G. Immunotherapy for ovarian cancer: what’s next? *J Clin Oncol*. 2011 Mar 1;29(7):925–33.

## Antibody therapy

In the last fifteen years, antibody-based therapies for hematologic cancers and solid tumors have become well-established therapeutic strategies. Following rituximab’s 1997 FDA-approval for treating chemotherapy-resistant non-Hodgkin lymphomas, 18 other molecular antibodies have gained FDA approval for use in oncologic care. Antibody therapy is a promising area of research and increasingly antibody therapies are being used in ovarian cancers.

### Bevacizumab

Bevacizumab (Avastin®, Roche) is a humanized monoclonal antibody (mAb) that binds to all isoforms of the vascular endothelial growth factor (VEGF) receptor ligand. VEGF is a key mediator of developmental angiogenesis and has been shown to regulate the vascularization of tumors [11]. Anti-VEGF antibody therapy has proven effective in multiple cancer subtypes including metastatic colorectal cancer, glioblastoma, non-small cell lung cancer, and renal cancers [12]. Ovarian cancer is a promising candidate for VEGF therapy; in biopsies from ovarian tumors, VEGF gene expression correlates with a poor prognosis [13]. The results of the phase III AURELIA trial show that the addition of bevacizumab to single-agent chemotherapy leads to increased progression-free survival (PFS) and overall response rate (ORR): PFS increased from 3.4 months to 6.7 months and ORR increased from 11% to 27% [14].

### Catumaxomab

Catumaxomab (Removab®, Fresenius Biotech GmbH) is a bispecific, trifunctional antibody directed against the epithelial cell adhesion molecule (EpCAM) and the T cell antigen CD3. Primary, metastatic, and recurrent epithelial ovarian cancers express EpCAM at a significantly elevated level compared to normal human surface epithelium [15]. Catumaxomab uses the EpCAM and CD3 binding domains to recruit and activate immune effector cells at the tumor site [16]. Antitumor effects are exerted via two complementary pathways. First, the bispecific structure of the antibody facilitates T cell-mediated cytotoxicity by localizing the T cell and tumor tissue. Simultaneously, the retention of a functional Fc domain on the bispecific antibody allows natural killer (NK) cells to lyse tumor via antibody-dependent, cell-mediated cytotoxicity [17,18]. In a randomized Phase II/III study including 129 patients, puncture-free survival was significantly longer in the group receiving catumaxomab [19]. Recently, a Phase II study of catumaxomab in chemotherapy-refractory ovarian cancer patient with malignant ascites demonstrated a benefit for catumaxomab therapy: catumaxomab prolonged both the puncture free interval and the time to first therapeutic puncture as well as producing improvement in quality of life [20].

### Cetuximab and panitumumab

Cetuximab (Erbitux®, BMS and Eli Lilly) is a chimeric, IgG1, mAb that binds to the extracellular domain of the epidermal growth factor receptor (EGFR) preventing EGFR-signaling and accelerating receptor internalization [21]. Cetuximab is routinely administered in the treatment of metastatic colorectal cancer and metastatic head and neck cancer. Up to 70% of ovarian cancer tumors and all histologic subtypes of ovarian cancer are EGFR-positive, making EGFR a promising therapeutic target in ovarian cancer [22]. *In vitro*, treating ovarian cancer cell lines with cetuximab inhibits cell growth, potentiates apoptosis, and impairs tumor metastasis [23].

A phase II trial of single-agent cetuximab in persistent/recurrent ovarian or primary peritoneal carcinoma found minimal clinical benefit: nine patients had stable disease, while only one of 25 patients achieved a partial response [24]. However, a phase II trial testing cetuximab in combination with carboplatin in platinum-sensitive ovarian cancer patients reported encouraging results [25]. Out of 29 patients, 9 demonstrated an objective response and 8 had stable disease. Panitumumab (Vectibix®, Amgen) is another anti-EGFR antibody with strong, early-phase clinical data. A phase II study of panitumumab and the chemotherapeutic pegylated liposomal doxorubicin (PLD) in platinum-resistant ovarian cancer reported a 9% partial response rate with stable disease in 19% of patients [26]. Out of the 32 evaluable patients, 24% had decreased levels of CA125, a surrogate marker for ovarian tumor burden.

The limitations of single-agent cetuximab, but success of anti-EGFR therapy in combination with chemotherapeutics highlights an important preclinical reality in immunotherapy: chemotherapy and immunotherapeutics can act synergistically to enhance antitumor immunity and improve therapeutic outcomes. Chemotherapy has historically been thought to be immunosuppressive, but recent work suggests the opposite. In a variety of tumor types, patients with greater numbers of TILs have a better clinical response to cytotoxic chemotherapy [27,28]. Oxaliplatin and cisplatin, common chemotherapies for ovarian cancer, have been shown to increase dendritic cells ability to induce antigen-specific T cell proliferation [29]. In ID8 murine ovarian cancer cells, the combination of chemotherapeutic PLD and the immunotherapy Interleukin 18 (IL-18) resulted in enhanced tumor suppression relative to either agent as a monotherapy [30]. In EGFR-expressing xenograft models, the efficacy of cetuximab can be increased by different classes of chemotherapeutic agents, including cisplatin, doxorubicin, paclitaxel and topotecan [31,32]. In the future, strategic implementation of chemotherapy-immunotherapeutic combinations could significantly improve ovarian cancer outcomes.

### TAM-targeting antibodies

Tumor-associated macrophages (TAMs) are a major stromal component in solid tumors. In the ovarian tumor microenvironment, TAMs are the most abundant subset of infiltrating immune cells [33]. However, macrophages are commonly classified into two subsets with different cytokine profiles, surface phenotypes, and functional effects on tumor growth [34]. Classical (M1-polarized) macrophages are activated by interferon-γ (IFN-γ) and characterized by the production of proinflammatory and immunostimulatory cytokines (e.g. IL-6, IL-12). M1 macrophages express high levels of major histocompatibility complex (MHC) I and II and thus play a critical role in tumor antigen presentation. Through immune system stimulation and antigen presentation, M1 macrophages have a net tumoricidal effect. Alternative (M2-polarized) macrophages are activated by Th2 cytokines (e.g. IL-4, IL-10) and exert anti-inflammatory effects. Recent work reports a correlation between improved 5-year prognosis and elevated M1 to M2 TAM ratio in ovarian cancer patients [35].

In ovarian tumors, M2 macrophages contribute to multiple mechanisms of TAM-mediated immunosuppression. Ovarian tumor macrophages upregulate and secrete the chemokine CCL22 which promotes T regulatory cell (Treg) trafficking to the tumor. In mice bearing primary human ovarian tumors, treatment with an anti-CCL22 mAb decreased Treg migration into tumors [36]. In addition to CCL22 expression, ovarian tumor macrophages express another receptor with immunosuppressive properties, B7-H4. B7-H4 is a member of the B7 family of T cell-antigen presenting cell (APC) regulatory molecules. In ovarian cancer patients, >70% of freshly isolated tumor macrophages expressed B7-H4 and *in vitro* B7-H4^+^ macrophages significantly decrease T cell proliferation and T cell activation upon TAA recognition [37]. Treatment of B7-H4^+^ TAMs with single chain fragments of antibody variable regions (scFvs) that target and block B7-H4, can reverse tumor immunosuppression and induce T cell activation [38].

While anti-CCL22 mAbs and anti-B7-H4 scFvs exert antitumor effects by modulating macrophage-T cell interactions, targeting the macrophage colony stimulating factor-1 receptor (CSF-1R) directly depletes immunosuppressive TAMs. Colony stimulating factor, also known as macrophage colony stimulating factor (CSF-1 or M-CSF), regulates the migration, proliferation, survival, and function of macrophages [39]. Macrophages rely on pro-growth, M-CSF signaling for survival and blocking CSF-1R provides an avenue for decreasing M2-polarized TAMs. In murine tumor models with high TAM-infiltration, the administration of an anti-CSF-1R mAb significantly reduced TAMs and simultaneously increased the ratio of cytotoxic CD8^+^ T cells to CD4^+^ T cells while decreasing the number of FoxP3^+^ Tregs [40]. In 2011, a humanized anti-CSF-1R mAb, RG7155 (Roche), entered clinical trials. The results from the ongoing Phase Ia/Ib clinical trial (NCT01494688) indicate that RG7155 treatment is well tolerated and effectively depletes TAMs [41]. Targeting macrophages is a promising therapeutic approach to ovarian cancer and encouraging early work indicates that CSF-1R blockade, anti-B7-H4 scFvs, and anti-CCL22 mAbs may generate potent antitumor responses.

## Immune checkpoint inhibitors

Immune checkpoints are inhibitory pathways that downregulate activated T cells following antigen presentation and costimulatory signaling by APCs. By controlling the intensity and duration of the immune response, immune checkpoint signaling prevents collateral self-tissue damage. During tumorigenesis, however, cancer cells express proteins that activate immune checkpoint pathways and induce immune suppression thereby evading targeting and removal by the immune system. The clinical successes of antibodies modulating immune checkpoints continue to fuel the enthusiasm surrounding immunotherapeutic approaches to cancer treatment.

### CTLA-4

The cytotoxic T-lymphocyte-associated protein 4 (CTLA-4 or CD152) plays a vital role in regulating T-cell activation [42]. Activation is triggered through antigen recognition by the T-cell receptor (TCR), but costimulatory and coinhibitory signaling dictates the magnitude of the resulting response. The cell surface molecule CD28 and its ligands CD80 (B7-1) and CD86 (B7-2) are the primary source of costimulatory signaling [43]. CD80 and CD86 are predominantly found on antigen-presenting cells like monocytes, activated B cells, and dendritic cells [44]. However, CD80 and CD86 do not exclusively induce activating signals, they are also the ligands of CTLA-4, a key negative regulator of T cell activation [45]. CTLA-4 directly competes with CD28 for binding to CD80 and CD86. CTLA-4 ligation results in the termination of T cell activation, cell cycle arrest, and T cell anergy. By limiting or reversing T cell activation, CTLA-4 serves as an important immune checkpoint that helps contain immune responses.

In the immunosuppressive tumor microenvironment, blocking CTLA-4 has the potential to directly activate CD4^+^ and CD8^+^ effector T cells, leading to tumor clearance. In a variety of preclinical tumor models, the administration of an antagonistic anti-CTLA-4 antibody induced tumor rejection [46]. The successes of anti-CTLA-4 therapy revitalized interest in the field of immunotherapy and resulted in the 2011 FDA approval of the anti-CTLA-4 mAb ipilimumab (Yervoy®, Bristol-Myers, Squibb) [47]. The majority of clinical experience with ipilimumab has come from studies in patients with melanoma, but a Phase II study of ipilimumab monotherapy in patients with platinum-sensitive ovarian cancer is ongoing (NCT01611558).

### PD-1 and PD-L1 axis

The programmed cell death protein-1 (PD1) and its ligand (PD-L1) represent a promising immune checkpoint pathway that can be targeted to reverse tumor-mediated immunosuppression. Ligation of PD1 suppresses the lytic activity of immune effector subsets [48]. In ovarian cancer, PD-L1 expression on monocytes in the ascites and blood of patients with malignant cancer correlates with poor clinical outcome [49]. Cytotoxicity assays revealed that PD-L1 overexpression on murine ovarian cancer ID8 cells inhibited cytotoxic T lymphocyte (CTL) degranulation and reduced CTL-mediated tumor lysis; PD-L1 blockade reversed this effect. Recently, results were presented from a phase I trial of the anti-PD1 mAb, nivolumab (BMS), in patients with platinum-resistant ovarian cancer [50]. Out of fifteen patients treated with nivolumab, 20% achieved partial responses and 26% had stable disease. The validation of antibodies targeting the PD-1/PD-L1 axis arrived in late 2014 when the FDA granted accelerated approval to pembrolizumab (Keytruda, Merck). Pembrolizumab is an anti-PD1 mAb that achieved an ORR of 26% in ipilimumab-refractory advanced melanoma patients [51]. mAbs targeting PD1 and PD-L1 are currently being evaluated in over 100 clinical trials and ovarian cancer remains a prioritized indication for testing.

### IDO

In addition to transmembrane receptor targets, metabolic enzymes are being investigated as therapeutic strategies for reversing immunosuppression within the tumor microenvironment. Indoleamine 2,3-dioxygenase (IDO) is the leading metabolic immune regulator in clinical development.

IDO is an intracellular enzyme that catalyzes the initial and rate-limiting step of the oxidative catabolism of the amino acid tryptophan [52]. Tryptophan catabolism is believed to influence immunodynamics via two mechanisms: tryptophan depletion starves T lymphocytes triggering cell cycle arrest and apoptosis and the tryptophan metabolite kynurenine is toxic to lymphocytes [53,54]. Kynurenine accumulation has been linked to the selective apoptosis of T cells, monocytes, and macrophages [55,56]. Kynurenine can also induce the expansion of Tregs. Plasmacytoid dendritic cells (pDCs) with elevated levels of IDO can convert naive CD4^+^ T cells into Treg cells [57]. Inhibiting IDO in pDCs abrogates Treg generation, but adding kynurenine restores the conversion of CD4^+^CD25^−^ T cells into Tregs. With mounting evidence for the immunosuppressive effects of IDO, its role in tumor immune evasion is increasingly under investigation.

The role of IDO in human cancer was first documented in 2003 by Uyttenhove *et al.* who reported constitutive IDO expression in most human tumors and linked elevated IDO levels to a low frequency of TILs in murine cancer models [58]. In ovarian cancers, immunohistochemical scoring of IDO expression in surgically resected tissue has demonstrated that IDO is prevalent in ~56% of ovarian tumors and correlates with a reduced number of CD8^+^ TILs [59]. IDO expression also inhibits NK cell accumulation in ovarian tumors and promotes tumor angiogenesis [60]. In patients with serous-type ovarian cancer, increased synthesis of IDO is positively associated with impaired survival, but this trend was not true for other histological subtypes of OC [61]. Gene expression profiling has also found elevated IDO levels in paclitaxel-resistant ovarian cancer tissues [62].

The first IDO-targeted therapy to enter preclinical testing was 1-methyl-tryptophan (1-MT), a small molecule inhibitor of IDO. In an IDO-overexpressing ovarian cancer model, combination treatment of paclitaxel and 1-MT synergistically prolonged mouse survival relative to paclitaxel monotherapy [59]. This result supports earlier work that suggests IDO-inhibition augments the efficacy of cytotoxic chemotherapeutic agents and IDO-based combination therapy may eradicate tumors that are refractory to single-agent therapy [63]. 1-MT treatment also significantly suppressed tumor dissemination and ascites after IDO-overexpressing ovarian cancer cells were implanted into syngeneic immunocompetent mice [64].

Currently, the IDO inhibitor Indoximod® (NewLink Genetics) is in five clinical trials with encouraging early-phase clinical data [65]. In a phase I trial of Indoximod in combination with docetaxel in patients with metastatic solid tumors 18% of treated patients exhibited a partial response and 41% had stable disease. In a phase IB/II trial of indoximod in combination with AD.p53DC, a dendritic cell cancer vaccine, 9% of patients achieved stable disease with initial treatment. Following combination therapy, 11 patients showed tumor progression and then received gemcitabine-based chemotherapy; 54% of these patients achieved an objective response. A second-generation IDO-inhibitor, NLG919 (NewLink Genetics), entered clinical trials in late 2013 (NCT02048709). Both inhibitors are orally bioavailable and are being tested in patients with recurrent solid tumors.

In addition to the IDO inhibitors developed by NewLink Genetics, INCB024360 (Incyte Corp.), an IDO1 inhibitor, has entered clinical testing. In preclinical data, INCB024360 was shown to significantly inhibit tumor growth and to induce T and NK cell proliferation and IFN-γ production [66]. In the Phase I safety and tolerability trial, at doses above 300 mg twice a day, 90% inhibition of IDO activity was observed, with pneumonitis and fatigue reported as the only dose-limiting toxicities [67]. A Phase II trial of INCB024360 monotherapy versus tamoxifen in patients with epithelial ovarian cancer is ongoing (NCT01685255). As IDO inhibitors enter the clinic, it will be important to monitor the immunologic effects of global IDO inhibition with special attention focused on the onset of autoimmune dysfunction. In the MRL-lpr mouse model of spontaneous lupus disease, 1-MT treatment accelerates lupus onset [68].

## Vaccine strategies

Therapeutic cancer vaccines have been an area of research interest since the 1920s, when injections of lymph node extracts were used to treat Hodgkin’s lymphoma. The promise of vaccine strategies is the potential to “teach” individual patients’ immune systems to recognize, target, and eradicate tumor cells in an approach that employs both adaptive and innate immunity. Vaccines aim to provoke a tumor-specific immune response by increasing TAA presentation by APCs thus generating tumor-antigen specific cytotoxic T lymphocytes. Unlike chemotherapy, surgery, radiotherapy, or antibody therapy, an effective vaccine-induced immune response could establish a state of immunological memory that persists after tumor clearance and indefinitely suppresses tumor regrowth. In recent decades, multiple approaches to therapeutic cancer vaccines have been developed and dendritic cell vaccines have emerged as efficacious in ovarian cancer.

### *Ex vivo* DC vaccines

Dendritic cells (DCs), the most potent class of APCs, are responsible for processing cancer antigens from tumor cells and presenting peptide fragments to naive T cells, B cells, and NK cells. DC vaccines attempt to enhance DC uptake and presentation of TAAs, galvanizing an antitumor response. In ovarian cancer, a promising TAA for DC vaccines is mucin 1 (MUC-1). MUC-1 is a heavily glycosylated, type 1 transmembrane protein that is overexpressed in a large number of cancers including colorectal, pancreatic, and ovarian [69]. While multiple MUC-1 vaccines are now in development, CVac® (Prima BioMed) is the leading candidate for treatment of ovarian cancer. CVac is produced by culturing isolated DCs *ex vivo* with MUC-1. In the CAN-003 Phase II study, 63 epithelial ovarian cancer patients in complete remission received CVac. In the patients who had achieved a remission after second-line therapy, PFS and OS were increased [70].

### *In vivo* DC vaccines

*Ex vivo* DC vaccines require isolating and stimulating DCs on a patient-specific basis and are thus costly, labor-intensive, and mostly limited to large, academic medical centers. An attractive alternative is to use an “off-the-shelf” therapeutic that is not patient-specific and stimulates DCs to uptake TAAs *in vivo.* One example of an early, preclinical success with this approach in ovarian cancer is the administration of a vaccine based on the MSLN-Hsp70 fusion protein [71]. The MSLN-Hsp70 protein combines a scFv to mesothelin (MSLN), an antigen overexpressed in pancreatic and ovarian tumors, and a heat shock protein from *Mycobacterium tuberculosis* (Hsp70). In this system, the dendritic cells are activated by the tuberculosis Hsp70 and, because the fusion protein localizes DC activation to MSLN-expressing cells, immediately recognize the tumor antigen. Administration of the bifunctional fusion protein in mouse models of ovarian cancer significantly enhanced survival and slowed tumor growth while augmenting tumor-specific CD8^+^ T cell responses.

### *Whole tumor* DC-based vaccines

A potential avenue for improving the efficacy of DC vaccines is the inclusion of multiple tumor antigens during DC priming. When immunotherapeutic intervention is limited to a single target antigen, there is the possibility of tumor escape. Tumor escape occurs when the tumor mutates to downregulate the immunogenic antigen and thus evades immune system surveillance and continues to proliferate. By preparing vaccines from whole tumor cells, the immune system can be trained to recognize a broad range of TAAs. Whole-tumor preparations also eliminate the need for researchers to identify an optimal antigen target; unknown tumor antigens may drive or contribute to the cellular immune response. Recent work utilized a lysate of tumor cells to demonstrate the effectiveness of whole tumor DC-based vaccination against ovarian cancer in both a preclinical and clinical setting [72]. In the clinic, out of the five patients who received the DC vaccine, two had PFS of 24 months or more (NCT01132014).

### Peptide vaccines

Peptide vaccines rely primarily on the immunogenicity of the injected peptides to stimulate an immune response. In the cancer setting, the peptides chosen for the vaccine are TAAs. Recently, overlapping long peptides (OLP) from NY-ESO-1 were used as a peptide vaccine in combination with two different adjuvant preparations: Montanide and Poly-ICLC [73]. After OLP vaccination alone, NY-ESO-1-specific CD8^+^ T cells and NY-ESO-1-specific antibody responses were undetectable, but after vaccination with OLP and administration of both Montanide and Poly-ICLC, 91% of patients demonstrated both a NY-ESO-1-specific CD8^+^ T cells and a NY-ESO-1-specific antibody response. Additional targets for peptide vaccine strategies in ovarian cancer include p53, Her-2/neu, and CA125 [74-76]. While peptide vaccines have proven effective at eliciting TAA-specific immune responses, combination with complementary immunotherapeutic modalities may be necessary to generate potent antitumor immunity.

### Recombinant viral vaccines

Recombinant viral vaccines utilize genetically modified viruses as vectors for introducing TAA-encoding DNA into cells within the body [77]. Viruses are an attractive antigen-delivery system because most viruses elicit an immune response; viral immunogenicity induces immune cell trafficking to the injection site, where professional APCs, like DCs, encounter the newly introduced TAAs. APCs then return to lymph nodes with the digested and expressed TAA and induce a tumor-specific humoral or cellular immune response. The discovery of TAAs for ovarian tumors has spurred the development of therapeutic recombinant viral vaccines for patients with ovarian cancer.

The cancer testis antigen NY-ESO-1 is a well-documented target for immunotherapy of ovarian cancer and has been the focus of multiple cancer vaccine studies [78-80]. Recently, NY-ESO-1-recombinant vaccinia and fowlpox vectors were used in parallel Phase II clinical trials, producing encouraging immunologic data [81]. Induction of a NY-ESO-1-specific antibody response was observed in 42% of patients who were seronegative at the baseline assessment. CD8^+^ T cell responses were induced in 32% of patients (14% had a preexisting response), while CD4^+^ T cell responses were induced in 22% of patients (68% had a preexisting response). In the ovarian cancer patients, the median PFS was 21 months and the median overall survival (OS) was 48 months. Poxviral vectors are also the basis for the PANVAC vaccine platform, which is actively being tested in ovarian cancer patients (NCT00088413). PANVAC is a cancer vaccine therapy that contains transgenes for the TAAs MUC-1 and carcinoembryonic antigen (CEA) as well as transgenes for three human T cell costimulatory molecules, collectively known as TRICOM (B7-1, intracellular adhesion molecule-1 and leukocyte function-associated antigen-3) [82]. In a pilot study of PANVAC in 14 ovarian cancer patients, median time to progression was 2 months and median OS was 15.0 months [83].

The convenience, low toxicity, and potential therapeutic activity of vaccine strategies make them an irresistible target of future immunotherapeutic research. As optimal vaccine platforms, antigen targets, and adjuvant conditions are identified, vaccines will become an increasingly valuable therapeutic option for treating ovarian cancer.

## Adoptive cell therapy

Adoptive Cell Therapy (ACT) is an immunotherapeutic technique that uses autologous or allogeneic antitumor lymphocytes to induce cancer regression. In autologous ACT, lymphocytes are isolated via apheresis, cultured, and assayed for tumor recognition. Highly reactive cultures are expanded and reinfused into the cancer patient. Lymphodepleting chemotherapy preceding infusion eliminates immunosuppressive cells and supports the *in vivo* survival and expansion of tumor-specific lymphocytes. First described in 1988, ACT initially demonstrated strong responses in melanoma, but has since been tested in other tumor types including ovarian cancers [84,85]. A 1995 trial comparing the effect of adoptive cell therapy on advanced-stage epithelial ovarian cancer found the 3-year overall survival rates between the ACT group and the controls, who did not receive ACT, to be 100% and 67.5%, respectively [86]. However, ACT is limited by the availability of tumor-specific lymphocytes. Recent advances in cellular genetic engineering have addressed this limitation. Using retro-viral vectors, antigen-specific T cell receptors are transduced into normal peripheral blood lymphocytes (PBLs) converting them into cells that accurately target and lyse tumor [87]. The antigen-specific T cell receptors, or chimeric antigen receptors (CARs), are composed of scFvs specific to the tumor antigen of interest and a T cell signaling domain capable of inducing activation.

In the ID8 mouse model of ovarian cancer, T cells transduced to express an NKG2D-based CAR demonstrated an endogenous antitumor immunity and long-term, tumor-free survival [88]. Surviving mice developed T cell memory responses that were protective: 90% of mice rejected the rechallenge with ID8 tumor cells. In a separate study, treatment with chimeric-NKG2D T cells also increased the number of host CD4^+^ and CD8^+^ T cells at the tumor site and increased the number of antigen-specific host CD4^+^ T cells in the tumor and draining lymph nodes [89]. With encouraging initial results of CAR-based therapy in ovarian models, multiple ovarian-specific tumor antigens are being used in CAR development and ACT strategies are moving towards the clinic [90].

## Combinatorial immunotherapy

While single-agent immunotherapies have produced promising clinical responses, unleashing the maximal antitumor immune response is likely to require combinatorial therapeutic strategies [91]. As illustrated above, for a tumor to uncontrollably proliferate and evade detection by the immune system, multiple “tumor escape mechanisms” must act concertedly [92]. By combining immunotherapies, different stages of tumor escape can be targeted, creating the possibility of synergistic and additive effects between agents [93]. Despite the novelty of immunotherapeutic treatments in ovarian cancer, combination strategies are an area of intense research and combinatorial trials have entered the clinic.

The initial combination immunotherapies for ovarian cancer patients were based on immune checkpoint blockade strategies. In the ID8-VEGF model of ovarian carcinoma, researchers observed that up to half of TILs were double positive for both CTLA-4 and PD-1 and displayed a decreased proliferation capacity and inability to produce effector cytokines [94]. Co-administration of anti-PD-1 antibodies and anti-CTLA-4 antibodies reversed the TIL dysfunction and induced tumor regression in 50% of the mice relative to 25% with either agent as a monotherapy. The addition of the GM-CSF gene vaccine, GVAX, to the therapeutic regimen further increased tumor rejection to 75% in the ID8-VEGF mice. The combination of anti-CTLA-4 and GVAX has also been tested in eleven-patients with metastatic ovarian carcinoma [95]. Three patients achieved stable disease as measured by CA-125 levels and one patient achieved an objective response by radiographic criteria and maintained disease control over four years with regular infusions of anti-CTLA-4 antibody. In addition to preclinical research, the recent successes of trials evaluating nivolumab have spurred nivolumab-based combinations, including a Phase I/II trial evaluating nivolumab and the IDO inhibitor INCB24360 in patients with ovarian neoplasms (NCT02327078).

Combination immunotherapy trials have also expanded to include vaccine strategies, mAb therapy, and ACT. In a trial for patients with recurrent ovarian cancer, a DC-based autologous whole-tumor lysate vaccine was tested in combination with the anti-angiogenic mAb bevacizumab (NCT01312376). Of the six patients who participated, four patients demonstrated a clinical benefit and an increase in tumor-reactive T cells following vaccination; tumor reactivity was quantified by measuring IFN-γ secretion [96]. From these four patients, three patients with residual measurable disease advanced to a study where they received adoptive transfer of autologous vaccine-primed, CD3/CD28-co-stimulated T cells (NCT00603460). By the end of study, one patient had achieved a complete response, one patient had a partial response, and one patient had progressive disease [96].

In the near future, the number of combination immunotherapy studies targeting ovarian cancer patients will dramatically increase. However, the application of multiple immunotherapies simultaneously requires careful considerations. Primarily, with combination treatments there is a potential for overlapping toxicities and elevated risk of sequela due to immune system disinhibition. Effective adverse-event management will be a crucial element of successful combination immunotherapy. Secondly, there is a need to optimize the timing of agent administration; in some situations, sequential administration may prove more advantageous than concurrent administration. If immune-related toxicities can be controlled and ideal dosing determined, combinatorial immunotherapy may dramatically improve the clinical outcomes of ovarian cancer patients.

## Conclusions

Early clinical successes have validated immunotherapeutic treatment strategies and immunotherapies hold immense potential to improve outcomes for patients with ovarian cancer. In future research, it will be important to identify the dominant immunosuppressive pathways within ovarian tumors. A better understanding of the relevant immuno-oncologic pathways and their corresponding biomarkers will allow patients to be optimally matched with therapies. In conjunction with biomarker research, combination strategies should continue to be explored. Combination approaches are uniquely appealing because different classes of immunotherapies target potentially synergistic stages of tumor immunity. Within the tumor microenvironment, immunotherapies may increase antigen release or antigen presentation, induce cytotoxicity via effector immune subsets, or remove immunosuppression. Identifying the optimal combination of drugs to provoke a concerted antitumor response will translate to substantial improvements in long-term clinical benefit.
